# Rural protein insufficiency in a wildlife-depleted West African farm-forest landscape

**DOI:** 10.1371/journal.pone.0188109

**Published:** 2017-12-13

**Authors:** Björn Schulte-Herbrüggen, Guy Cowlishaw, Katherine Homewood, J. Marcus Rowcliffe

**Affiliations:** 1 Zoological Society of London, Institute of Zoology, London, United Kingdom; 2 University College London, Department of Anthropology, London, United Kingdom; Cornell University College of Veterinary Medicine, UNITED STATES

## Abstract

**Introduction:**

Wildlife is an important source of protein for many people in developing countries. Yet wildlife depletion due to overexploitation is common throughout the humid tropics and its effect on protein security, especially for vulnerable households, is poorly understood. This is problematic for both sustainable rural development and conservation management.

**Methods:**

This study investigates a key dimension of protein security in a cash-crop farming community living in a wildlife-depleted farm-forest landscape in SW Ghana, a region where protein–energy malnutrition persists. Specifically, we monitored protein sufficiency, defined as whether consumption met daily requirements, as benchmarked by recommended daily allowance (RDA). We focus on whether more vulnerable households were less likely to be able to meet their protein needs, where vulnerability was defined by wealth, agricultural season and gender of the household head. Our central hypothesis was: (a) vulnerable households are less likely to consume sufficient protein. In the context that most plant proteins were home-produced, so likely relatively accessible to all households, while most animal proteins were purchased, so likely less accessible to vulnerable households, we tested two further hypotheses: (b) vulnerable households depend more on plant protein to cover their protein needs; and (c) vulnerable households are less likely to earn sufficient cash income to meet their protein needs through purchased animal sources.

**Results:**

Between 14% and 60% of households (depending on plant protein content assumptions) consumed less than the RDA for protein, but neither protein consumption nor protein sufficiency co-varied with household vulnerability. Fish, livestock and food crops comprised 85% of total protein intake and strongly affected protein sufficiency. However, bushmeat remained an important protein source (15% of total consumption), especially during the post-harvest season when it averaged 26% of total protein consumption. Across the year, 89% of households experienced at least one occasion when they had insufficient income to cover their protein needs through animal protein purchases. The extent of this income shortage was highest during the lean season and among poorer households.

**Conclusions:**

These findings indicate that despite wildlife depletion, bushmeat continues to make a substantial contribution to protein consumption, especially during the agricultural lean season. Income shortages among farmers limit their ability to purchase bushmeat or its substitutes, suggesting that wildlife depletion may cause malnutrition.

## Introduction

The widespread overexploitation of wildlife for food or income in tropical countries has pushed forests with previously abundant wildlife into a wildlife-depleted state, leading to the “empty forests syndrome” [1–3]. While this has generally been considered a conservation problem, the common dependence of rural communities on wildlife suggests that a loss of such resources may also have implications for human well-being, unless resource users are able to find appropriate substitutes [4–6].

The hunting of wild animals for food or income (bushmeat) is an important source of animal protein and livelihood in rural communities around the world [7]. It also acts as a safety net, contributing to income- and consumption-smoothing, both in vulnerable households throughout the year and in all households during times of vulnerability, e.g., the agricultural lean season [8,9]. Thus, children in remote areas with abundant wildlife populations in the Congo Basin display lower levels of stunting than those in areas with less abundant wildlife [6], and it is estimated that the loss of wildlife from predominantly bushmeat-dependent communities in rural Madagascar would result in a 29% increase in the numbers of children suffering from anaemia, and a tripling of anaemia cases among children in the poorest households [10]. Similarly, for urban and peri-urban sites in the Amazon, households consuming bushmeat had higher levels of iron, zinc, and vitamin C than those that did not, and simply substituting bushmeat with chicken would lead to a 65% reduction in iron, 24% reduction in zinc and 17% reduction in vitamin C [11]. These cross-regional observations suggest a causal link between bushmeat supply and human health in rural areas in the humid tropics. Understanding the linkages between wildlife depletion and protein sufficiency, and the ability of consumers to substitute bushmeat with alternative dietary protein, is therefore important for both conservation and human well-being.

Rural- and urban consumption studies give insights about the ability of consumers to diversify their diet and switch from bushmeat to alternative sources of animal protein. Bushmeat is widely consumed in both urban and rural areas of sub-Saharan Africa. However, the per capita consumption of bushmeat, as well as its contribution to protein intake, is generally higher in rural areas [12] and is inversely related to market access [13,14]. The key distinction between rural and urban areas lies in the price and availability of bushmeat and its substitutes, such as livestock and fish. Generally, urban consumers have access to a wide range of meat/fish types and the high price of bushmeat makes it an expensive commodity that is frequently consumed only by a wealthy minority [15–17]. In contrast, bushmeat is relatively inexpensive in rural areas, often hunted by the consumers themselves, and alternatives are both less readily available and generally more expensive than bushmeat [12,18]. Within rural communities, bushmeat consumption is often highest among the poorest households [12,13], highlighting its role as a safety net for the poor, who can least afford to purchase bushmeat substitutes. Among agricultural communities, this effect is often most critical during the lean season, when household cash income is low and food prices peak [19–21].

With few exceptions (see [9,10]), the consequences of declining wildlife populations for rural communities in sub-Saharan Africa, especially for those households utilising bushmeat as a safety net, are largely unknown. Observations from wildlife-depleted areas in Southeast Asia suggest that rural communities can make the dietary switch from bushmeat to domesticated meat or fish, provided that (1) households have access to cash income generating activities other than bushmeat hunting, and (2) cash income levels are sufficient to cover protein needs through purchasing meat/fish [22]. However, it is uncertain whether the same applies to wildlife-depleted forest communities in west and central Africa where a) agricultural incomes may not be sufficient to replace hunting profits, and b) the availability of fish inland and local livestock production are both limited [23].

The present study was conducted in rural Ghana, where both wildlife depletion [24,25] and malnutrition [26] are widespread. The first signs of wildlife depletion in Ghana were evident in the 1960s [27] and have since resulted in the widespread depletion of target species [24,25]. Malnutrition has long been and remains endemic in Ghana. The WHO Nutrition Landscape Information system gives the following national-level data on the nutritional status of children aged 0-5yrs for Ghana in 2014: 11% underweight; 19% stunted and 5% wasted [28]. The most recent WHO data for Ghana’s Western Region, where the present study was located refer to 2011 (close to the 2008–9 study period) and record 17% of rural children as underweight, 30% stunted and 10% wasted [29]. Malnutrition thus remains prevalent at both national and regional levels in Ghana.

Within this broader setting, we seek to assess whether a forest community in tropical Africa, and in particular the most vulnerable households within that community, can adapt to depleted wildlife populations or otherwise face protein malnutrition. To do this, we evaluated protein sufficiency, defined as whether overall levels of protein intake met the recommended daily allowance. We then investigated whether protein sufficiency decreased with household vulnerability, where vulnerability was defined along three dimensions: 1) seasonality, whereby all households were considered vulnerable during the agricultural lean season (compared to the main harvest season) [30]; 2) wealth, whereby households with a low participatory wealth rank were considered more vulnerable than households of a high wealth rank [31]; and 3) gender, with female-headed households considered more vulnerable than male-headed households [32]. Specifically, we hypothesised that: (H1) vulnerable households consume less protein (including bushmeat) and consequently show lower protein sufficiency. Given prior expectations that most plant proteins would be home-produced, so likely relatively accessible to all households, while animal proteins would be scarcer, mostly purchased, and so likely less accessible to vulnerable households, we tested two further hypotheses:; (H2) the contribution of bushmeat and other meat/fish to protein consumption is lower, and the contribution of plant protein is higher, in vulnerable households; and (H3) vulnerable households are less likely to earn sufficient cash income to meet their protein needs through purchased animal sources.

## Methods

### Ethics statement

The research was carried out in accordance with the ethics guidelines of the Association of Social Anthropologists of the UK and Commonwealth (ASA) and the methods, including the consent procedure, were approved by the Department of Anthropology Ethics Committee, University College London. Prior to the data collection a meeting was held with all community members to carefully explain the purpose of the study and obtain informed oral consent of the research participants—written consent was not possible due to the low level of literacy in the area. Subsequently, BSH spent several months in the village prior to the study to familiarise himself with local livelihoods and pilot the questionnaire (see S1 Questionnaire.). Meetings with informants on a one-to-one basis were used to explain the research objectives, answer any questions raised and confirm oral consent. Discussions with informants were documented to ensure oral consent was confirmed by all households participating in the survey. All data were anonymised to reduce the risk of harm to informants. The research was associated with a project of the Zoological Society of London operating in a nearby area and was covered by its research permits obtained from relevant authorities.

### Study site

This study was carried out in the village of Wansampobreampa (hereafter Wansampo) (6.06°N, -2.73°W) in the Akontombra district, Western Region, SW Ghana. The village lies within the northern part of the Upper Guinea forests, a global biodiversity hotspot [33]. The region has experienced a strong decline in wildlife populations due to a combination of habitat destruction and high hunting pressure [25]. The study community is located inland more than 100km away from the coast and situated within a mosaic of intensively managed cocoa farms and timber production forest (the Sui Forest Reserve) both showing high levels of wildlife depletion [24]. As a result, most animal protein consumed was purchased, resulting in household expenditures of US$0.92/day [21].

Cocoa farming is the main livelihood activity within the community, although households engage in a variety of economic activities: 59% of household cash income is earned from on-farm activities (sales of cocoa beans, food crops and livestock, and farm labour) and the remainder from a combination of off-farm business and labour (31%), cash gifts (5%), trade in non-timber forest products (2%) and other miscellaneous income sources (3%) [34]. The community has about 350 people living in 70 households. The village is bisected by a laterite road that connects two district capitals (Sefwi Wiawso and Akontombra) and is accessible all year round. The frequent traffic of passenger cars facilitates transportation to district markets.

### Data collection

As described in detail previously [21], the study took place over a period of twelve months (July 2008 to June 2009) including the ‘lean season’ when average cocoa income was at its lowest (July to September 2008), the main agricultural season (hereafter ‘harvest season’: October 2008 to January 2009) and the period in-between (hereafter ‘post-harvest season’: February to June 2009). These three seasons were determined using data on cocoa sales obtained from the community (for further details see [34]) and followed the classifications of seasons described for cocoa growers in other parts of Ghana [19,35]. Prior to the survey period, BSH spent six months in the village piloting the questionnaire, familiarising himself with local livelihoods, and establishing relationships with the villagers to ensure high data quality [36]. Semi-structured questionnaires were used to assess the harvest and use of bushmeat, household production and expenditure, and the consumption of meat and fish. Each household was interviewed about once per month using 24-hour recall periods. A total of 787 complete interviews were used in this study, covering 63 households (the remaining seven households were either not permanent residents or reluctant to participate in the research). All interviews were conducted in Twi/Sefwi, by BSH or an international field assistant, with help from local assistants. All assistants received extensive training in social research methods prior to data collection. The mean exchange rate was US$1.0 = 11,862 Cedis (June 2008 to June 2009; http://www.oanda.com/).

### Household demography and wealth

Household demographic information was collected during a census in August 2007 and revised during two further censuses (April 2008 and June 2009), recording information on the household head, number of household members, their age and education. Repeated assessments were required since household composition varied strongly throughout the data collection period and was in many cases ambiguous during the first and second censuses. Data from the third census benefited from the prolonged observation period in the village, particularly from dinner surveys recording household members present, providing an in-depth understanding of relationships between households and dependencies. The household demographic data included in the models were derived from the final census, as it was considered of the highest quality.

Participatory household wealth ranking exercises were conducted with seven long-standing community members of different genders, socio-economic backgrounds and community neighbourhoods. All households were grouped into four wealth categories based on their mean participatory wealth score [37]. Outcomes of the wealth ranking were cross-checked against independent wealth characteristics (household expenditure and house roof value) and were strongly correlated [34].

### Estimating protein consumption

Meat/fish and plant protein surveys were part of the comprehensive monthly socio-economic household survey that also collected detailed information on monetary and non-monetary incomes and expenditures. This allowed the use of a multi-step cross-checking process (triangulation) to improve the data quality of notoriously difficult consumption surveys [38].

Protein consumption estimates were derived by first eliciting the monetary value of the meat/fish and food crops consumed and subsequently converting this to protein consumption (in g/AME/day, where AME refers to adult male equivalent and is explained in detail below). All conversion factors used are outlined below. While recall interviews could be considered less accurate than estimates based on weighed food consumption, they are much less intrusive and time consuming for respondents, and are widely used in the natural resources literature (eg [39]) as well as in national household surveys (eg [40]). Converting from value to weight is likely to introduce some error into the measurement process, however our experience of people’s behaviour and recall ability leads us to believe that these errors were small relative to actual variation across households, and there is furthermore no reason to expect systematic bias that could obscure the expected patterns of consumption. The detailed triangulation data verification steps applied during the research are outlined below.

### Animal protein

Animal protein consumption was defined as any meat, fish or other animal product consumed by household members during the 24 hours prior to an interview. Unless individual animal protein types are explicitly stated, meat/fish refers collectively to bushmeat, fish, crustaceans, molluscs and livestock.

Surveys elicited the type and monetary value (recorded in Cedis) of meat/fish consumed and grouped these into breakfast, lunch and dinner, depending on the time of consumption. These data were then cross-checked with data on wildlife harvest, household expenditure and gift income for the same period. Gift exchange surveys frequently recorded meat/fish meals received from or sent to non-household members and these were respectively added or subtracted from the household’s own consumption. Finally, the data were compared with information from dinner participant surveys, in which the identity of household members consuming dinner at and/or outside the household, and non-household members consuming dinner at the household, were recorded. The value of meat/fish consumed by household members eating elsewhere was added to the household consumption and the value of meat/fish consumed by non-household members sharing the focal household meal was subtracted from the meat/fish available to household members. The consumption surveys were facilitated by the fact that people rarely stored meat/fish and most purchased or produced meat/fish was consumed on the same day. Also, most meat/fish was purchased from traders in the community and prices were readily available.

Meat consumption in Cedi/household/day was converted into protein consumption in gram/household/day. This was done using species-specific estimates of the price per kg body weight, derived from estimates of the monetary value per animal recorded during surveys in combination with body weight estimates from this study and the literature [41] (see S1 Table for details). Where no species-specific price per kg was available, the mean value of the taxonomic group was used, e.g. for an unidentified fish species the mean value across different fish taxa was used. Livestock data were based on local prices for animals of different age classes and mean weights obtained from Armbruster & Peters [42] and National Research Council [43]. Dressed meat weights were estimated as 65% of the original weight for bushmeat [39], 60% for goats and sheep, 70% for chicken [44], and 100% for fish and crustaceans, as no part of the animal was discarded (this study). The protein weight was calculated by multiplying the dressed weight with the respective protein content, which was 28% for bushmeat and 19% for fresh fish [39], 20% for chicken, 18% for goat and beef, 17% for sheep, 12% for pig, and 47% for dried fish [44]. We note milk and eggs as potential sources of animal protein but highlight that neither of these were recorded during interviews or observed during a prolonged period spent in the community.

### Plant protein

Estimates of plant protein available for consumption were derived from data on food crop production, purchases and gift exchange rather than from consumption surveys. This consumption proxy was justified on the basis that households harvested food crops on a daily basis and commonly consumed these within 24 hours. The analysis focused on the six most common food crops, namely plantain (*Musa paradisiaca*), cassava (*Manihot esculenta*), cocoyam (*Colocasia esculenta*), white yam (*Dioscorea alata*), yam (*Dioscorea* spp.) and okra (*Abelmoschus esculentus*). These are widely stated as the most important types of plant protein consumed in rural communities in SW Ghana [45].

To obtain the dressed weight of food crops, 31 surveys were conducted and the weight of the raw plant and the plant weight that went into the pot were recorded. This was done for cassava (mean percentage discarded = 33%; SD = 12%, N = 16), plantain (mean percentage discarded = 43%; SD = 5%, N = 11) and cocoyam (mean percentage discarded = 45%; SD = 21%, N = 4) showing that on average around 40% of the raw weight was discarded. The dressed weight of food crops was therefore defined as the raw weight minus 40%.

The two most common forms in which plant protein was consumed was as “ampesi” (boiled cocoyam or plantain) and “fufu” (a staple made of cassava and plantain). The sales price stated for “ampesi”corresponded to the value of the ingredients and could easily be converted into the protein weight equivalent. However, the stated price of fufu was higher than the cost of the ingredients and corresponded to prices in a local chop bar. This reflected the hard manual labour (pounding) involved in preparing fufu. To determine the conversion factor from recorded fufu price to protein weight, 29 independent surveys recorded the local sales price of the fufu meal and the amount and price of plantain and cassava used as ingredients. The estimated conversion factors were 0.19 for plantain and 0.26 for cassava (i.e., 19% and 26% of the fufu sales price was the price of raw plantain and cassava, respectively).

Food crops were grouped into two categories based on their protein content: low protein (including starchy staples such as yam, cassava and plantain), and high protein (including beans and groundnuts). Literature estimates of protein contents for low-protein food crops vary between 0.5% and 4.0% of wet weight, with most estimates lying between 1% and 2% (S2 Table). Utilising this range of estimates avoids the spurious sense of accuracy that can be associated with using single point estimates and also makes allowance for the variation in protein content caused by 1) ripening processes, reported to be a difference of 50% for unripe and ripe plantain [46], and 2) different preparation methods, that can also alter protein content to varying degrees [47]. Lower and upper limits of 1% and 2% protein content were therefore used throughout the analysis. However, while estimates of protein consumption and protein security were substantially lower in the 1% than 2% scenario, all models using either scenario came to the same conclusions and highlighted the same variables as strong predictors. Hence, we decided to show only one scenario (2% protein content) here and have provided the detailed model outputs for the alternative 1% protein content scenario in the supplementary materials (cited in text). In contrast to the low-protein content foods, the level of variation in the protein content estimates of high-protein food crops was substantially lower (S2 Table). Their protein contents were approximated as 22% and 25% for beans and groundnuts/groundnut butter, respectively.

Interviews were conducted after people had returned from their farm, which meant that farm produce was often still present during the interviews and could be weighed. Where crop weight data were available these were used instead of verbal estimates of the harvest value. Plant protein destined for sale or gift was excluded from the analysis.

### Assessing protein sufficiency

Protein sufficiency was assessed by comparison of total protein consumption rates (animal and plant protein) against a threshold of 0.75g per kg human body weight per day, which has been proposed as the recommended daily allowance (RDA) of protein consumption [48]. For an adult male or female (these are not distinguished) with an average body weight of 70kg this means a protein consumption of 52.5g/day. The choice of protein RDA is controversial for several reasons. First, the optimal level of protein intake for maximal health and function remains uncertain [49] resulting in a number of different RDAs being proposed [48–50] and these RDAs are continually being revised. Second, different people have different protein requirements. For example, Bauer et al. [51] suggested that those who are physically active or suffer from acute or chronic diseases have higher protein needs (1.2–1.5 g/kg/d). Similarly, lactating women and children may have higher protein needs [52]. Given the high level of uncertainty and variation in protein needs we decided to use a single estimate that is commonly used in the protein security literature related to natural resources and bushmeat [4,14,39,53–56]. This approach provides consistency and facilitates comparison across studies. We also stress that the RDA adopted is generally lower than other RDA estimates and hence results in a conservative estimate that limits the chances of overestimating protein insufficiency.

Household composition was converted to adult male equivalent (AME) units, following [57]. Estimates of AME/household were assessed for each interview based on the presence of household members during the main meal, i.e., dinner. This dynamic approach to household composition reflects strong temporal variation in cocoa farmers’ households as production and consumption units [58,59]. The average household comprised 3.11 AMEs (SD = 1.54; range = 0.79–8.16, N = 63).

It is important to note that the estimates of animal and plant protein consumption reported here did not control for within-household variation in protein consumption, which can be strongly skewed towards adult males [60], but instead refer to the total amount of animal protein consumed by, and the amount of plant protein available to, the household as a whole.

### Household income

Monetary incomes included irregular sources in the form of either small frequent payments (e.g., from daily labouring or small trade), or larger but less frequent payments (mainly from cocoa sales), as well as regular salaries. During each interview, three different recall periods were therefore used to record incomes. 1) Frequent, small, irregular payments were captured by eliciting all monetary household incomes obtained within 24 hours prior to an interview. 2) Larger but less frequent irregular payments were defined as those exceeding 50,000 Cedis (US$ 4.22), and were captured using two-week recall, divided by 14 to estimate daily income. This threshold payment size was judged appropriate for large infrequent incomes and was confirmed as such by informants. 3) Finally, interviewees earning a regular income from employment were asked about their monthly salary and this was divided by 30 to obtain the mean daily income. Income sources were recorded carefully to avoid double counting across recall periods. All incomes used in this analysis are gross incomes as these were the best approximation of the money available to a household at the time of an interview.

### Data analysis

All statistical analyses were performed in the R environment, version 2.9.2 [61]. To explore the relationship between a response variable and independent variables, Generalized Linear Mixed Models (GLMM) were used (‘lme4’ package, version 0.999375–32 [62]), including household as a random effect in all cases.

The analyses focused on seven household response variables. For hypothesis 1, these comprised two response variables: the total daily protein consumption (g/AME/day), and protein sufficiency, i.e., whether protein consumption exceeded the RDA (2 factor levels: yes/no). For hypothesis 2, these comprised four response variables: the percentage of total protein consumed coming from food crops, low-protein food crops, fish, and bushmeat. For hypothesis 3, these comprised one response variable: the likelihood that 50% of the household gross income exceeded the cost of reaching the RDA through animal protein purchases (2 factor levels: yes/no). In all seven cases, three predictor variables (fixed effects) and their potential interactions were assessed: the participatory household wealth (4 factor levels), the agricultural season (3 factor levels: harvest season/post-harvest season/lean season), and the gender of the household head (2 factor levels: female (FHH)/male (MHH)). All analyses were based on aggregated means per household (n = 63) per season (n = 3), and the total sample size was 185 (results were not obtained for four of the potential 189 household seasons).

To control for the potentially confounding effects of household demographics and composition, four additional variables were included as fixed effects in the models: the number of active (aged 16 to 65 years) male household members (continuous variable, range: 0–4), the household dependence ratio (ratio of dependent to total number of household members, range: 0–0.75 subdivided into four groups with thresholds at >0.2, >0.4 and >0.6), the age of the household head (range: 21–84 years subdivided into six groups with thresholds at >30, >40, >50, >60 and >70), and the household head level of education (number of years in formal education (range: 0–12 years subdivided into five groups with thresholds at 0; 1 to 3; 4 to 6; 7 to 9; >9).

Model evaluation was based on the information-theoretic approach using Akaike’s Information Criterion (AIC) to infer the relative support for alternative models [63]. The interpretation of GLMM results was based on two criteria. First, the i’th model’s relative support was evaluated with reference to the model with the lowest AIC value using the AIC difference: ΔAIC_i_ = AIC_i_ − AIC_min_. Models with ΔAIC_i_ ≤ 2 were deemed to have substantial support, those with 4≤ ΔAIC_i_ ≤7 considerably less support, and ΔAIC_i_>7 essentially no support [64]. Secondly, the ΔAIC of the null model (hereafter ΔAIC_N_) provided a measure of the relative confidence in the interpretation of the results. If support for the null model was relatively high (ΔAIC_N_ ≤ 2), then confidence in the alternative models was reduced, even if the best model was not the null model.

All interpretations of relative support for individual variables were further triangulated by assessing the respective effect sizes and standard errors and the ΔAIC_i_ of the univariate model. The validity of models, regarding the assumed normal distribution of within-group errors and randomly distributed random effects, were tested qualitatively by plotting within-group residuals that provide a good surrogate for within-group errors, and inspection of fitted versus residual plots [65].

## Results

### Contribution of different protein sources

Protein derived from food crops and animals contributed 53% and 47% to total protein consumption, respectively (assuming 2% protein content for staple crops, hereafter ‘2% scenario’; Table 1). However, the contribution of protein sources to total protein consumption was strongly dependent on the assumption about the protein content of staple crops. Applying a 1% protein content (hereafter ‘1% scenario’), reduced the contribution of food crops and turned meat/fish into the main source of protein, i.e., 38% and 62% of total protein consumed, respectively. Overall, the majority of protein was derived from three sources: dried fish, plantain and cassava were most frequently consumed and comprised between 55% and 61% of total protein consumed (for 1% and 2% scenarios, respectively).

**Table 1:** Average daily meat/fish and food crop consumption in Wansampo (N = 185).

	%	Dressed g/AME/day ^a^		Protein g/AME/day	
Type	Consumption frequency ^b^	Mean	Median (range)	Mean	Median (range)
**Meat/fish**	**93.3**	**103**	**86 (0–376)**	**32**	**27 (0–116)**
**Fish**	74.6	50	39 (0–319)	19	16 (0–71)
Fish (dried)	62.6	32	25 (0–131)	15	12 (0–61)
Fish (fresh)	14.0	16	0 (0–277)	3	0 (0–52)
Fish (tinned)	3.3	2	0 (0–47)	<1	0 (0–9)
**Bushmeat**	32.3	39	26 (0–256)	10	7 (0–71)
Mammals	24.6	29	15 (0–256)	8	4 (0–71)
Snails	3.0	2	0 (0–49)	<1	0 (0–5)
Other	7.3	7	0 (0–107)	2	0 (0–29)
**Livestock**	14.2	13	0 (0–265)	2	0 (0–48)
Beef	8.3	4	0 (0–70)	1	0 (0–13)
Chicken	2.9	4	0 (0–155)	1	0 (0–31)
Goat	1.5	2	0 (0–133)	<1	0 (0–24)
Sheep	1.3	1	0 (0–70)	<1	0 (0–12)
Pig	0.9	1	0 (0–47)	<1	0 (0–6)
Other	0.1	<1	0 (0–33)	<1	0 (0–13)
**Unknown** ^c^	5.2	1	0 (0–38)	<1	0 (0–18)
**Food crops**	**84.1**	**1,712**	**1,316 (0–11,510)**	**38**	**29 (0–238)**
**Staple crop (2%)**	79.2	1,697	1,316 (0–11,480)	34	26 (0–230)
Plantain	62.4	679	420 (0–5,524)	14	8 (0–111)
Cassava	62.2	633	462 (0–5,954)	13	9 (0–119)
Cocoyam	13.1	153	0 (0–5,461)	3	0 (0–109)
Cocoase	11.8	133	0 (0–3,030)	3	0 (0–61)
Okra	9.1	80	0 (0–4,400)	2	0 (0–88)
Yam	1.5	20	0 (0–2,347)	<1	0 (0–47)
**Beans (>20%)**	23.1	15	4 (0–551)	3	1 (0–122)
Groundnut	19.3	7	0 (0–77)	2	0 (0–19)
Beans	4.2	8	0 (0–546)	2	0 (0–120)

^a^ dressed weight for meat/fish is shown. For food crops this refers to wet weight minus 40% skin weight (see Methods for details)

^b^ % of interviews recording the consumption of meat/fish or food crops

^c^ interviewees stated meat/fish but no further details

The consumption of meat/fish averaged 103g/AME/day in dressed weight and 32g/AME/day in protein weight. The main source of animal protein was fish (predominantly from wild sources); consumed in 75% of interviews and comprised 58% of animal protein and 27–37% of total protein consumed (2% and 1% scenario, respectively). Dried fish was the most prominent type of fish consumed (recorded in 63% of interviews).

Bushmeat was the second main source of animal protein. Bushmeat consumption was recorded in 32% of interviews and averaged 39g/AME/day in dressed meat weight and 10g/AME/day in protein weight, corresponding to 31% of animal protein and 14–19% of total protein consumed (2% and 1% scenario, respectively). Livestock was a minor source of protein supply (6% of animal protein and 3–4% of total protein, 2% and 1% scenario, respectively) and mainly consumed during celebrations such as Christmas.

The consumption of food crops was substantially higher than the consumption of meat/fish, averaging 1.7kg/AME/day dressed weight. The main sources of plant protein were plantain and cassava averaging 1.3kg/AME/day (68%-71% of plant protein and 26%-39% of total protein, 1% and 2% scenario, respectively). Hence, despite their low protein content, consumption of a large amount enabled people to derive a substantial proportion of their total protein intake from these crops. The consumption of “fufu”, a traditional meal prepared from plantain and cassava, played a key role in this pattern. Fufu was consumed for dinner on a daily basis and a common saying was “if you have not eaten fufu, you have not eaten that day”. Small quantities of meat/fish were occasionally consumed for breakfast and lunch (without fufu) but the bulk of animal protein was served for dinner (with fufu). In fact, daily meat/fish consumption was perceived as a necessity, as “without meat or fish you cannot eat fufu“, which meant not eating at all that day.

In contrast, crops with high protein content such as beans and groundnuts, contributed only marginally to total protein consumption (2% and 3% of total protein consumed, respectively). Groundnuts were not farmed by households in the village, instead small quantities were purchased and mainly used to flavour soups rather than as a source of protein or a substitute to animal protein. Alternative soups were a light vegetable soup and palm oil soup. Beans were used infrequently as they were not a traditional crop in the area and had to be bought at the district market.

### Protein consumption and sufficiency

The first analysis investigated patterns of protein consumption and sufficiency, and their relationship to household vulnerability (H1). Mean total protein consumption varied strongly across households and depending on the assumption about the protein content of ‘low protein’ food crops, ranging from 53g/AME/day to 70g/AME/day for the 1% and 2% scenario, respectively (Table 2). In the conservative 1% scenario, over half of all households failed to achieve the RDA, while in the more optimistic 2% scenario, most households reached the RDA but a substantial minority (14%) still failed to consume sufficient protein.

**Table 2:** Average protein consumption and percentages of households consuming sufficient protein across households, relative to two benchmarks: A recommended daily allowance of 52.5 g per adult male equivalent per day, and a higher threshold of 100 g/AME/d (percentages do not add up to 100% due to rounding).

	Plant protein content	
	1% scenario	2% scenario
Mean consumption	53 (g/AME/day)	70 (g/AME/day)
%hhs <52.5g/AME/day	60%	14%
52.5g > %hhs <100g/AME/day	33%	52%
%hhs >100g/AME/day	6%	32%

Despite the variation in protein sufficiency between households, there was little support from the GLMMs for any effect of household vulnerability (wealth, seasonality, or gender of the household head) on either daily protein consumption per AME or protein sufficiency (ΔAIC_N_ = 0 for both). However, total household protein consumption increased with household size (S3 Table, S1 Fig) and wealthier households had higher AME values (no effect was found for gender of the household head and seasonality) (S4 Table, S2 Fig). This highlights that wealthier households consumed more protein but due to larger household size, the consumption per AME did not vary substantially across wealth categories.

### Determinants of the contribution of protein sources

We then tested the hypothesis that vulnerable households derived a larger proportion of their total protein consumption from certain food types compared to less vulnerable households (H2), testing all food crops (i.e., total plant protein), “low-protein” food crops, fish, and bushmeat, in turn. In the first case, vulnerability was a strong determinant of plant protein consumption, although not always in the direction predicted: seasonality received most support from the GLMM (Table 3), but mainly due to the larger contribution of plant protein during both the harvest season (55% of total protein, SE = 2%) and lean season (53% of total protein, SE = 3%) compared to the post-harvest season (45% of total protein, SE = 3%), rather than during the lean season only or the lean and post-harvest season. However, there was some evidence that plant protein contributed more to households in the three lower wealth categories (52% of total protein, SE = 3%) than to the wealthiest households (44% of total protein, SE = 3%) and for animal protein we found the opposite pattern with 8% higher animal protein consumption among wealthiest households. Support for an effect of the gender of the household head was lower (only appearing in one of the two models with ΔAIC ≤2) and this was confirmed by the minor difference (4%) in plant protein consumption observed between female- and male-headed households.

**Table 3:** Results of GLMM analysing the effect of participatory household wealth (wealth), gender of the household head (gender) and seasonality (season) on the contribution of plant protein within a household’s total protein consumption (assuming 2% protein content of low-protein food crops; for corresponding GLMM results assuming 1% protein content, see S5 Table). Mean consumption estimates per household per season were analysed (N = 185).

Model	Delta AIC	Akaike weight
wealth+season	0	0.37
wealth+gender+season	0.7	0.26
season+gender	2.9	0.09
season	3.0	0.08
wealth+gender*season	3.2	0.07
wealth	4.7	0.04
season*gender	5.4	0.03
wealth+gender	5.4	0.02
wealth*season	7.3	0.01
null	7.5	0.01
gender	7.6	0.01
wealth*season+gender	8.0	0.01
wealth*season+gender*season	10.1	<0.01

**Fig 1:**
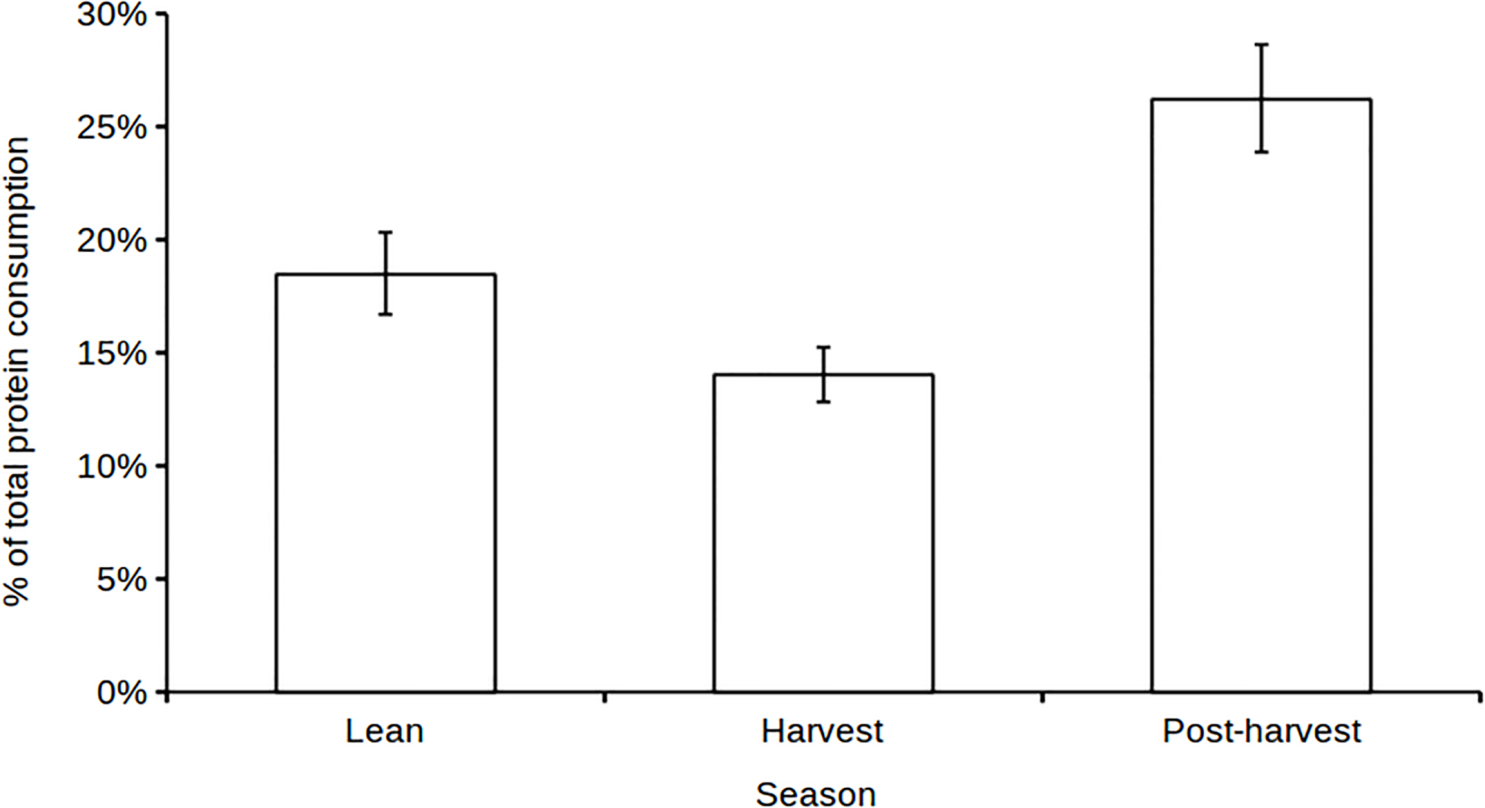
The percentage of total protein derived from bushmeat consumption for households that consumed bushmeat (scale of the response) across seasons. Standard errors are shown.

Although this study was unable to find strong evidence for an effect of household vulnerability on the relative contribution of plants to protein consumption, it remained possible that vulnerability had a stronger effect on the main sources of protein, namely”low-protein”food crops, fish, and bushmeat. However, neither “low protein” food crop nor fish consumption co-varied with household vulnerability (wealth, gender of the household head, or season: ΔAIC_N_ = 1.8 in both cases). Nevertheless, among households that consumed bushmeat, its contribution to total protein consumption varied strongly across seasons. It was lowest during the harvest season, intermediate during the lean season and highest during the post-harvest season (Fig 1 & Table 4). There was little evidence for an effect of household wealth or gender of the household head on relative bushmeat consumption.

**Table 4:** Results of GLMM analysing the effect of household wealth (wealth), gender of the household head (gender) and seasonality (season) on the contribution of bushmeat protein for households that consumed bushmeat (scale of the response) (assuming 2% protein content of low-protein food crops; for corresponding GLMM results assuming 1% protein content, see S6 Table). Analysed were mean consumption estimates per household per season (N = 136).

Model	Delta AIC	Akaike weight
season	0	0.59
season+gender	1.7	0.25
wealth+season	4.2	0.07
season*gender	4.8	0.05
wealth+gender+season	6.1	0.03
wealth+gender*season	9.4	0.01
null	11.0	<0.01
gender	12.7	<0.01
wealth*season	14.0	<0.01
wealth	15.6	<0.01
wealth*season+gender	16.0	<0.01
wealth+gender	17.6	<0.01
wealth*season+gender*season	19.2	<0.01

### Does income limit meat/fish consumption?

Finally, to test the hypothesis that animal protein consumption is limited by household income (H3) we assessed whether household income was sufficient to cover daily protein requirements through the purchase of animal protein. Daily gross income averaged US$7.15/household (SD = 15.08), equating to US$ 2.73/AME (SD = 5.40). The animal protein source with the lowest price per unit of protein that was commonly available in Wansampo was a type of dried fish, locally referred to as herring (US$ 8.7/kg protein, see S1 Table; other protein types were cheaper but not readily available at the site). For an average household with 3.1 AME (SD = 1.7) the cost of covering the RDA for protein (162.8g) through purchasing dried fish protein was US$ 1.41/household/day. Purchasing a combination of more expensive yet still commonly available fish types (US$ 11.9/kg protein) increased the cost to US$ 1.94/household/day, while the cost for covering protein needs through buying the most commonly available type of bushmeat (giant pouched rat, US$ 9.1/kg protein), was similar to dried fish (US$ 1.48).

**Fig 2:**
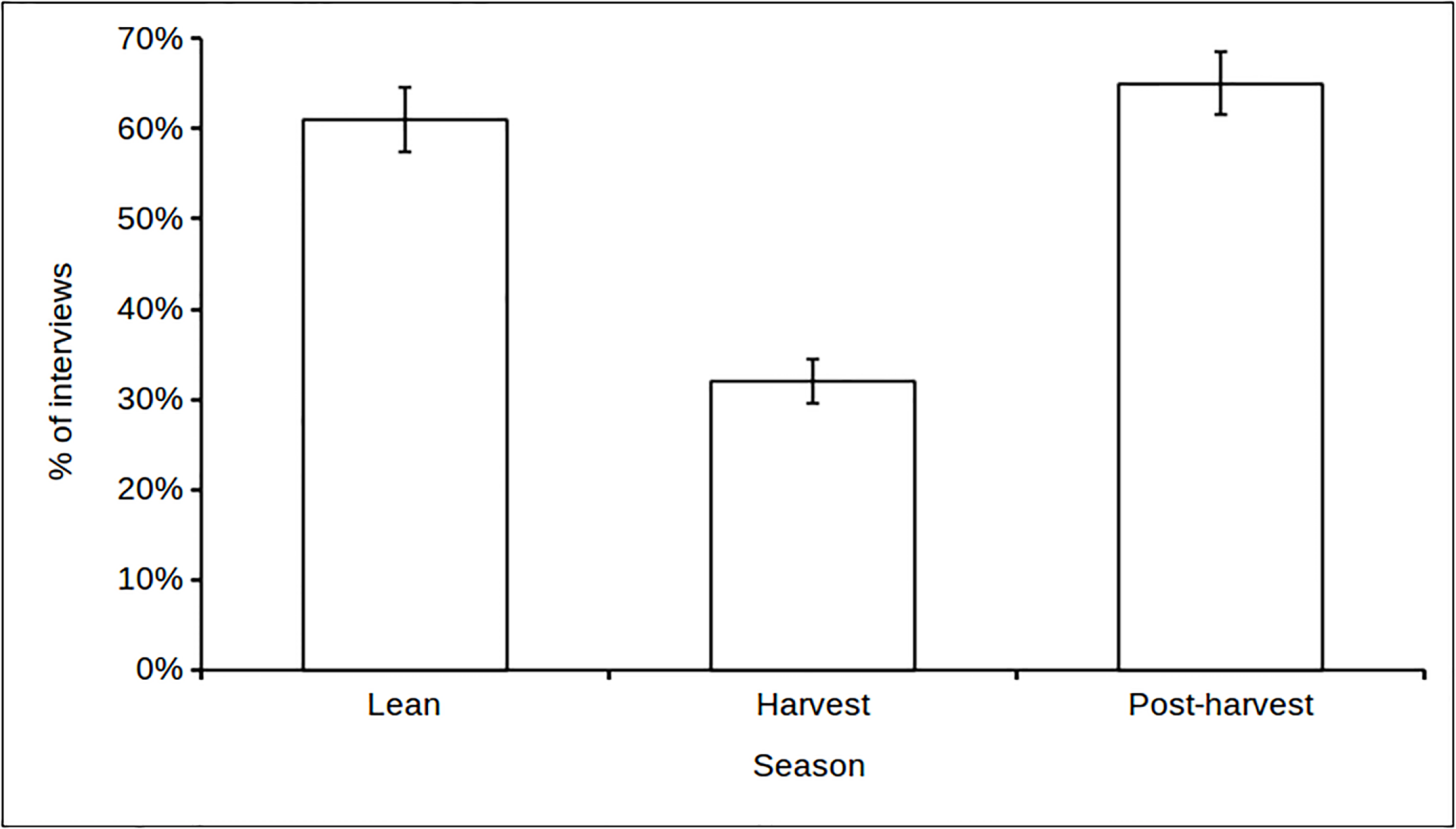
The percentage of interviews with insufficient gross cash income (50%) to cover the RDA through purchase of the cheapest animal protein source available across seasons. Standard errors are shown.

To assess a household’s ability to purchase sufficient animal protein, we compared the total daily gross cash income with the cost of animal protein necessary to achieve the RDA from animal protein alone. Insufficient income levels were recorded among 89% of households at least once and in a total of 35% of interviews. To take into account that households incurred other important expenditures, and therefore could not spend all of their income on buying meat/fish, we considered the likelihood that households would be able to purchase sufficient animal protein to meet their daily protein requirements with 50% of their total gross income. We note that setting a 50% threshold is arbitrary and merely aims to assess whether households would be able to cover their protein needs through animal protein purchases utilising a large share of their available income. The frequency with which this was not possible, i.e., the level of income insufficiency, was strongly seasonal (Table 5). Specifically, the level of income shortage was highest during the lean and post-harvest seasons and lowest during the harvest season (Fig 2). In addition, there was some indication that income shortages were highest among the poorest groups of households (bottom two groups: 52%, SE = 2%) compared to the wealthier groups of households (top two groups: 44%, SE = 3%). Rerunning the model with the top two and bottom two wealth groups merged resulted in the model including both season and wealth receiving most support (Table 6). In addition, both models (Tables 5 and 6) provide some support for gender of the household head, despite the means for male- (49%, SE = 2%) and female-headed households (48%, SE = 3%) being nearly identical. Evidence for the lack of support for gender of the household head is provided by the respective univariate models (Tables 5 and 6) that in both cases performed worse than the null model, suggesting that the main support for models with ΔAIC_i_<2 was due to the variables season and wealth. We conclude that animal protein consumption may be limited by household cash income (H3), and that this effect is strongest outside the harvest season and among poor households.

**Table 5:** Results of binomial GLMM assessing the likelihood of 50% of household gross income exceeding the amount needed to purchase animal protein > RDA in relation to household wealth (wealth), gender of the household head (gender) and seasonality (season).

Model	Delta AIC	Akaike weight
season	0	0.35
wealth+season	0.5	0.28
season+gender	1.5	0.16
wealth+gender+season	2.0	0.13
season*gender	4.3	0.04
wealth+gender*season	4.8	0.03
wealth*season	9.5	<0.01
wealth*season+gender	11.0	<0.01
wealth*season+gender*season	13.6	<0.01
Null	55.5	<0.01
wealth	55.7	<0.01
gender	57.2	<0.01
wealth+gender	57.3	<0.01

**Table 6:** Results of binomial GLMM assessing the likelihood of 50% of household gross income exceeding the amount needed to purchase animal protein > RDA in relation to household wealth (wealth, top two and bottom two wealth categories combined), gender of the household head (gender) and seasonality (season).

Model	Delta AIC	Akaike weight
wealth+season	0.0	0.39
season	1.5	0.18
wealth+gender+season	1.6	0.17
season+gender	3.0	0.09
wealth*season	3.4	0.07
wealth+gender*season	4.6	0.04
wealth*season+gender	5.1	0.03
season*gender	5.8	0.02
wealth*season+gender*season	8.0	0.01
wealth	55.5	0.00
null	57.1	0.00
wealth+gender	57.2	0.00
gender	58.7	0.00

## Discussion

In this study, we explored patterns of protein insufficiency in Wansampo, a West African farming community situated in a wildlife-depleted landscape. We found moderate to high levels of protein insufficiency through the year (14% to 60% of households, respectively, depending on alternative assumptions about plant protein content). However, we found no evidence that protein insufficiency could be predicted by household vulnerability, where vulnerability was assayed by wealth, agricultural season, and gender of the household head. The contribution of fish or low-protein food crops in the diet was similarly independent of household vulnerability, in contrast to the consumption of all food crops and bushmeat. Finally, we found evidence that cash income was inadequate to purchase sufficient protein to meet the recommended daily allowance among 89% of households in at least one interview. Unlike protein sufficiency, the adequacy of household cash income was correlated with household vulnerability.

We surprisingly failed to find evidence for a correlation between household vulnerability and protein sufficiency (contrary to hypothesis H1), despite the fact that wealthy households consumed more protein at the household level, due to variation in household sizes: AME increased with household wealth, so consumption per AME remained constant across wealth groups. Such mediating effects of household size on protein consumption patterns [17,66] and nutritional status [67] have been observed before. Nevertheless, protein sufficiency was also unaffected by our two other vulnerability indicators (seasonality and gender of the household head) and there was no evidence to suggest that household size had a mediating effect on them. Further research is required to understand the mediating effect of household size on household vulnerability and protein security as well as the effect of food sharing between households. However, a possible explanation lies in the substitution of different protein types between households of high or low vulnerability, which we will discuss below.

With respect to the consumption of different types of animal and plant protein (hypothesis 2), the consumption of meat/fish was considered a necessity in the Wansampo community. Interviewees frequently stated that meat/fish was an essential ingredient, albeit in small quantities, of their main meal. However, since bushmeat was only harvested by a small number of individuals [21] and livestock was rarely eaten, most animal protein had to be purchased. This meant that a substantial share of the daily household expenditure was spent on buying meat/fish [21]. This resembles findings from Equatorial Guinea, where rural communities spent a larger part of their disposable income on meat/fish purchases than urban households, primarily due to the higher costs of animal protein and lower income in rural areas [15]. This led the authors to suggest that the observed differences in the relative cost of animal protein explained the existence of a wealth-related effect on protein consumption in urban areas and its absence in rural areas.

Variation in the consumption of different sources of animal protein due to price differences has been documented in various studies assessing the cross-price elasticity of demand for bushmeat in relation to substitutes [68–70]. Similarly rational behaviour that is sensitive to the high price of meat/fish in Wansampo may explain why households consumed relatively small amounts of animal protein compared to plant protein. It may also provide a further explanation of why vulnerability had no substantial effect on protein sufficiency, i.e. poorer households were able to maintain levels of protein consumption that were comparable to wealthier households by consuming larger amounts of plant protein, an explanation that is in line with the observed effect of wealth on plant protein consumption. The consumption of large amounts of meat/fish was considered a luxury even for the wealthiest households and limited to festivities, such as Christmas or the birth of a child. During such occasions, livestock would be slaughtered and large amounts of meat were consumed. Most households owned livestock but since animals were primarily kept for special occasions, they contributed little to protein consumption and were only a minor source of income [21].

In contrast to meat/fish, low-protein food crops were mostly obtained from a household’s own production and did not incur substantial monetary cost. With a low protein-to-calory ratio, they were a cheap source of food that served to satisfy hunger. However, their nutritional value is poor, with low levels of vitamins and micronutrients [71]. This classes them as”unsatisfactory food” especially for children [72,73], raising questions about the nutritional status of the community beyond protein security. Bushmeat is considered an important source of micro-nutrients (reviewed in [56]) and Golden *et al*. [10] provide evidence for the negative effects of reduced bushmeat consumption if not substituted by alternatives. Yet, even substituting bushmeat by the same amount of chicken may result in a substantial decrease in iron, zinc, and vitamin C content in the diets [11]. While our study focused on protein security, we acknowledge the importance of bushmeat as a source of micro-nutrients and highlight the need for further work on this topic, particularly in wildlife-depleted areas.

The contribution of bushmeat to protein consumption was lowest during the harvest season, when income was highest, but peaked when vulnerability was intermediate, i.e., post-harvest season, rather than during the lean season. This finding neither fully rejects nor confirms our hypothesis. However, the peak in the contribution of animal protein coincided with a peak in the importance of bushmeat consumption, reflecting increased hunting activity and reduced sales of bushmeat during the same period, leaving more bushmeat for household consumption [21].

Overall, this analysis presents a complex pattern of household vulnerability effects on the consumption of different protein types that neither fully rejects nor confirms hypothesis 2. However, these results do provide further evidence for the role of bushmeat as a safety net during times of income shortage in the study community (e.g. [19,20]).

When we investigated the effect of cash income on household ability to meet the protein RDA (hypothesis 3), we did indeed find that cash income might limit protein consumption, with the greatest shortfall occurring during the lean season and to some extent among households of low wealth rank. Overall, a third of interviews recorded insufficient income to cover the RDA, which may have prevented these households from purchasing adequate amounts of animal protein. This finding suggests that in communities like Wansampo there may be insufficient income to substitute the bushmeat harvest with the purchase of alternative animal protein—an important prerequisite for protein sufficiency in a wildlife-depleted landscape. More broadly, it also questions the assumption that income earned through integration into the market economy compensates adequately for the loss of wildlife populations [22].

On the other hand, income shortages did not aggravate protein insufficiency in the community, as indicated by the absence of effects of seasonality or household wealth. This suggests that households adapted their protein consumption pattern to the highly seasonal element of their livelihoods. Our analysis suggests that this was achieved by a) a high dependence on low-protein food crops that were available year-round at basically no monetary cost, and b) minimising meat/fish expenditures to a level that could be afforded all year round. This risk-minimisation strategy may explain the absence of major effects of seasonality, wealth or household headship on protein consumption.

### Implications for policy and research

The protein consumption patterns observed, and the presence of protein insufficiency within the Wansampo community, raise a number of important implications for bushmeat policy, management and research. First, with an average plant protein consumption of 20-38g/AME/day, food crops can ameliorate the effects of wildlife depletion on protein security and should receive greater attention in the bushmeat literature, which currently focuses on meat and fish consumption. Second, the generally low consumption of animal protein and especially bushmeat in this community raises the potential for malnutrition due to insufficient micro-nutrient uptake. With the generally high level of wildlife depletion in the Ghanaian forest zone, there is the potential for similar patterns to occur across the region, and further research is needed to assess their generality and associated levels of malnutrition. Third, the recorded levels of protein insufficiency, and insufficient income to purchase meat/fish highlight the need to improve accessibility of affordable alternative protein sources in rural communities, especially those living in wildlife-depleted landscapes. Given the multiple constraints on supplying alternatives, it has been suggested that achieving sustainable bushmeat harvests may be the most pragmatic option for simultaneously promoting biodiversity conservation, food security and local livelihoods [5]. Wildlife depletion generally coincides with the transition of rural forest communities from a traditional subsistence economy to a cash economy. In the case of the study community, this process historically resulted in the development of a monoculture cash-crop farming system that is poor habitat for wildlife [24]. Yet many fast-reproducing wildlife species able to sustain high hunting pressure can occur in farmland, provided that adequate sources of food and shelter are available [12]. Diversification of cocoa monocultures into a mixed farming system, which would provide such food and shelter [74], has the prospect of increasing the supply of bushmeat while at the same time providing a source of cash income. Such mixed farming systems could also set aside areas for the cultivation of high-protein food crops, such as beans and groundnut, that are currently consumed only in small quantities in the community.

## Supporting information

S1 Table Sales prices for animals consumed or sold in Wansampo. (PDF)

S2 Table Literature estimates of food crop protein content. (PDF)

S3 Table Results of GLMM assessing the effect of household size (AME) on protein consumption per household. (PDF)

S4 Table Results of GLMM assessing the effect of household wealth (wealth), gender of the household head (gender) and seasonality (season) on household size (in AME). (PDF)

S5 Table Results of GLMM analysing the effect of participatory household wealth (wealth), gender of the household head (gender) and seasonality (season) on the contribution of plant protein within a household’s total protein consumption (assuming 1% protein content of low-protein food crops). Mean consumption estimates per household per season were analysed (N = 185).
(PDF)

S6 Table Results of GLMM analysing the effect of household wealth (wealth), gender of the household head (gender) and seasonality (season) on the contribution of bushmeat protein for households that consumed bushmeat (scale of the response) (assuming 1% protein content of low-protein food crops). Analysed were mean consumption estimates per household per season (N = 136).
(PDF)

S1 Fig Effect of households size (AME) on household protein consumption (g) (GLMM results in S3 Table). (PDF)

S2 Fig Effect of households wealth rank on household size (GLMM results in S3 Table). Standard errors are shown.
(PDF)

S1 Questionnaire Questionnaire used for the data collection. (PDF)
