# Supplementary material for: Rural protein insufficiency in a wildlife-depleted West African farm-forest landscape
Source: PLoS One. 2017 Dec 13;12(12):e0188109. doi: 10.1371/journal.pone.0188109 (PMC5728563; doi:10.1371/journal.pone.0188109)
Supplement: S1 Table — (PDF) [file pone.0188109.s001.pdf]

S1 Table. Sales prices for animals consumed or sold in Wansampo.

**Taxa**	**US$/animal ± SD**	**US$/kg (dressed)**	**US$/kg (protein)**	**N**
**Bushmeat mammals**				
African civet	21.08±5.96	5.85	21.29	2
Brush-tailed porcupine	7.39±2.34	4.40	15.99	17
Pel's anomalure	3.22±0.6	3.35	12.18	19
Common cusimanse	2.11±0.42	2.70	9.83	3
Giant pouched rat	1.65±0.46	2.50	9.09	255
Giant squirrel	1.31±0.54	2.83	10.28	2
Grasscutter	5.86±3.32	1.47	5.34	11
Lesser spot-nosed monkey	11.59±9.35	5.85	21.29	4
Maxwell's duiker	25.29±4.62	5.27	19.16	6
Slender mongoose	2.11±0.42	5.85	21.29	3
Bosman's potto	2.25±1.06	3.12	11.35	6
Royal antelope	6.43±3.22	4.76	17.31	4
Squirrel	0.98±0.49	5.47	19.89	25
Tree hyrax	4.04±1.48	2.24	8.16	31
Tree pangolin	3.71±1.14	2.58	9.38	42
**Bushmeat-other**				
Ahanta francolin	1.21±0.78	4.20	20.98	3
Crab	0.07±0.03	1.44	7.39	42
Crayfish	0.11±0.09	1.25	6.40	3
Monitor lizard	4.07±2.23	1.62	5.88	6
Snail	0.24±0.26	2.96	29.63	71
Tortoise	1.26±0.61	2.63	9.57	72
**Livestock**				
Beef[a]	N/a	3.96	22.02	20
Chicken[b]	5.11±1.17	7.09	35.45	27
Goat[b]	24.85±10.13	3.19	17.70	25
Pork[a]	N/a	2.99	24.94	15
Sheep[b]	33.08±10.7	2.80	16.46	33
**Fish**				
Tilapia (dried)	0.24±0.18	4.76	10.13	26
Herring (dried)	0.16±0.04	4.08	8.68	19
Mudfish (dried)	0.18±0.12	8.43	17.94	12
Fish (not dried)	0.49±0.11	2.06	10.95	11

Fish (tinned)[c]	N/a	6.00	31.91	7

[a] based on market data in Sefwi Dwenasi and consumption data in Wansampo due to insufficient data

[b] based on livestock survey in Wansampo using mean value per adult animal

[c] refers to tinned sardines and tuna; conversion factors were estimated from price per kg data
