# Supplementary material for: Rural protein insufficiency in a wildlife-depleted West African farm-forest landscape
Source: PLoS One. 2017 Dec 13;12(12):e0188109. doi: 10.1371/journal.pone.0188109 (PMC5728563; doi:10.1371/journal.pone.0188109)
Supplement: S2 Table — (PDF) [file pone.0188109.s002.pdf]

S2 Table. Literature estimates of food crop protein content.

Species	% protein	Source
**Low protein crops**		
Cassava	0.5	[1]
	0.6	[2]
	1.0	[3]
	1.0	[4]
	1.2	[5]
	1.4	[6]
	1.5	[7]
	4.0	[8]
Plantain	0.9	[3]
	1.1	[2]
	1.3	[6]
	1.7	[9]
	3.0 – 3.5	[10]
Yam	1.5	[6]
	1.5	[2]
	2.0	[3]
	2.0	[1]
Cocoyam	3.0 – 5.5	[11]
	10.4	[12]
Okra	2.0	[6]
	2.8	[2]
**High protein crops**		
Groundnut	22.6	[6]
	22.8	[2]
	23.0	[13]
	25.8	[2]
	28.0	[14]
Beans	22.0	[13]

References for S2 Table

1. Bradbury JH, Holloway WD. Chemistry of Tropical Root Crops. Canberra: Australian Centre for International Agricultural Research; 1988.

2. Food Standards Agency. McCance & Widdowson's the Composition of Foods Integrated Dataset [Internet]. London: HMSO; 2002. Available: http://tna.europarchive.org/20110116113217/http://www.food.gov.uk/multimedia/spreadsheets/cofids.xls

3. FAO. Roots, tubers, plantains and bananas in human nutrition. Rome: FAO; 1990 p. 198. Report No.: 24.

4. Gomez G, Valdivieso M. The effect of variety and plant age on cyanide content, chemical composition and quality of cassava roots. Nutr Rep Int. 1983;27: 595–865.

5. Ayankunbi MA, Keshinro OO, Egele P. Effect of methods of preparation on the nutrient composition of some cassava products-Garri (eba), "Lafun" and "Fufu." Food Chem. 1991;41: 349–354. doi:10.1016/0308-8146(91)90059-W

6. Saxholt E, Christensen AT, Moller A, Hartkopp HB, Hess Ygil K, Hels OH. Danish Food Composition Databank, revision 7 [Internet]. Copenhagen: Department of Nutrition, National Food Institute, Technical University of Denmark; 2008. Available: http://www.foodcomp.dk/

7. ITTA. Cassava in tropical Africa: a reference manual. Ibadan, Nigeria: IITA; 1990.

8. Ceballos H, Sanchez T, Chavez AL, Iglesias C, Debouck D, Mafla G, et al. Variation in crude protein content in cassava (Manihot esculenta Crantz) roots. J Food Compos Anal. 2006;19: 589–593. doi:10.1016/j.jfca.2005.11.001

9. Giami SY, Alu DA. Changes in composition and certain functional properties of ripening plantain (Musa spp., AAB group) pulp. Food Chem. 1994;50: 137–140. doi:10.1016/0308-8146(94)90110-4

10. Ketiku AO. Chemical composition of unripe (green) and ripe plantain (Musa paradisiaca). J Sci Food Agric. 1973;24: 703–707. doi:10.1002/jsfa.2740240610

11. Sefa-Dedeh S, Agyir-Sackey EK. Chemical composition and the effect of processing on oxalate content of cocoyam Xanthosoma sagittifolium and Colocasia esculenta cormels. Food Chem. 2004;85: 479–487. doi:10.1016/S0308-8146(02)00244-3

12. Hussain M, Norton G, Neale RJ. Composition and nutritive value of cormels of Colocasia esculenta (L.) Schott. J Sci Food Agric. 1984;35: 1112–1119. doi:10.1002/jsfa.2740351010

13. FAO. Agriculture, food and nutrition for Africa: a resource book for teachers of agriculture. Rome: FAO; 1997.

14. Lusas EW. Food uses of peanut protein. J Am Oil Chem Soc. 1979;53: 425–430.
