# Supplementary material for: Rural protein insufficiency in a wildlife-depleted West African farm-forest landscape
Source: PLoS One. 2017 Dec 13;12(12):e0188109. doi: 10.1371/journal.pone.0188109 (PMC5728563; doi:10.1371/journal.pone.0188109)
Supplement: S3 Table — (PDF) [file pone.0188109.s003.pdf]

S3 Table. Results of GLMM assessing the effect of household size (AME) on protein consumption per household.

**Model**	**Delta AIC**	**Akaike weight**
ame	0	1.00
null	29.33	<0.001
