# Supplementary material for: Rural protein insufficiency in a wildlife-depleted West African farm-forest landscape
Source: PLoS One. 2017 Dec 13;12(12):e0188109. doi: 10.1371/journal.pone.0188109 (PMC5728563; doi:10.1371/journal.pone.0188109)
Supplement: S4 Table — (PDF) [file pone.0188109.s004.pdf]

S4 Table. Results of GLMM assessing the effect of household wealth (wealth), gender of the household head (gender) and seasonality (season) on household size (in AME).

**Model**	**Delta AIC**	**Akaike weight**
wealth	0	0.94
gender	6.03	0.04
null	9.73	>0.01
season	11.56	>0.01
