# Supplementary material for: Rural protein insufficiency in a wildlife-depleted West African farm-forest landscape
Source: PLoS One. 2017 Dec 13;12(12):e0188109. doi: 10.1371/journal.pone.0188109 (PMC5728563; doi:10.1371/journal.pone.0188109)
Supplement: S5 Table — Mean consumption estimates per household per season were analysed (N = 185). (PDF) [file pone.0188109.s005.pdf]

S5 Table. Results of GLMM analysing the effect of participatory household wealth (wealth), gender of the household head (gender) and seasonality (season) on the contribution of plant protein within a household's total protein consumption (assuming 1% protein content of low-protein food crops). Mean consumption estimates per household per season were analysed (N=185).

**Model**	**Delta AIC**	**Akaike weight**
wealth+season	0	0.38
wealth+gender+season	0.8	0.26
wealth	3.2	0.08
season+gender	3.8	0.06
wealth+gender*season	3.8	0.06
season	3.9	0.05
wealth+gender	4.1	0.05
season*gender	6.8	0.01
gender	7.0	0.01
null	7.1	0.01
wealth*season	7.3	0.01
wealth*season+gender	8.0	0.01
wealth*season+gender*season	10.6	<0.01
