# Supplementary material for: Rural protein insufficiency in a wildlife-depleted West African farm-forest landscape
Source: PLoS One. 2017 Dec 13;12(12):e0188109. doi: 10.1371/journal.pone.0188109 (PMC5728563; doi:10.1371/journal.pone.0188109)
Supplement: S6 Table — Analysed were mean consumption estimates per household per season (N = 136). (PDF) [file pone.0188109.s006.pdf]

S6 Table. Results of GLMM analysing the effect of household wealth (wealth), gender of the household head (gender) and seasonality (season) on the contribution of bushmeat protein for households that consumed bushmeat (scale of the response) (assuming 1% protein content of low-protein food crops). Analysed were mean consumption estimates per household per season (N=136).

**Model**	**Delta AIC**	**Akaike weight**
season	0	0.59
season+gender	1.7	0.25
wealth+season	4.0	0.08
season*gender	5.4	0.04
wealth+gender+season	6.0	0.03
wealth+gender*season	9.8	<0.01
null	10.9	<0.01
gender	12.7	<0.01
wealth*season	14.2	<0.01
wealth	15.4	<0.01
wealth*season+gender	16.2	<0.01
wealth+gender	17.4	<0.01
wealth*season+gender*season	20.0	<0.01
