# Supplementary figures and images for: Rural protein insufficiency in a wildlife-depleted West African farm-forest landscape
Source: PLoS One. 2017 Dec 13;12(12):e0188109. doi: 10.1371/journal.pone.0188109 (PMC5728563; doi:10.1371/journal.pone.0188109)

S1 Fig. Effect of households size (AME) on household protein consumption (g) (GLMM results in S3 Table).

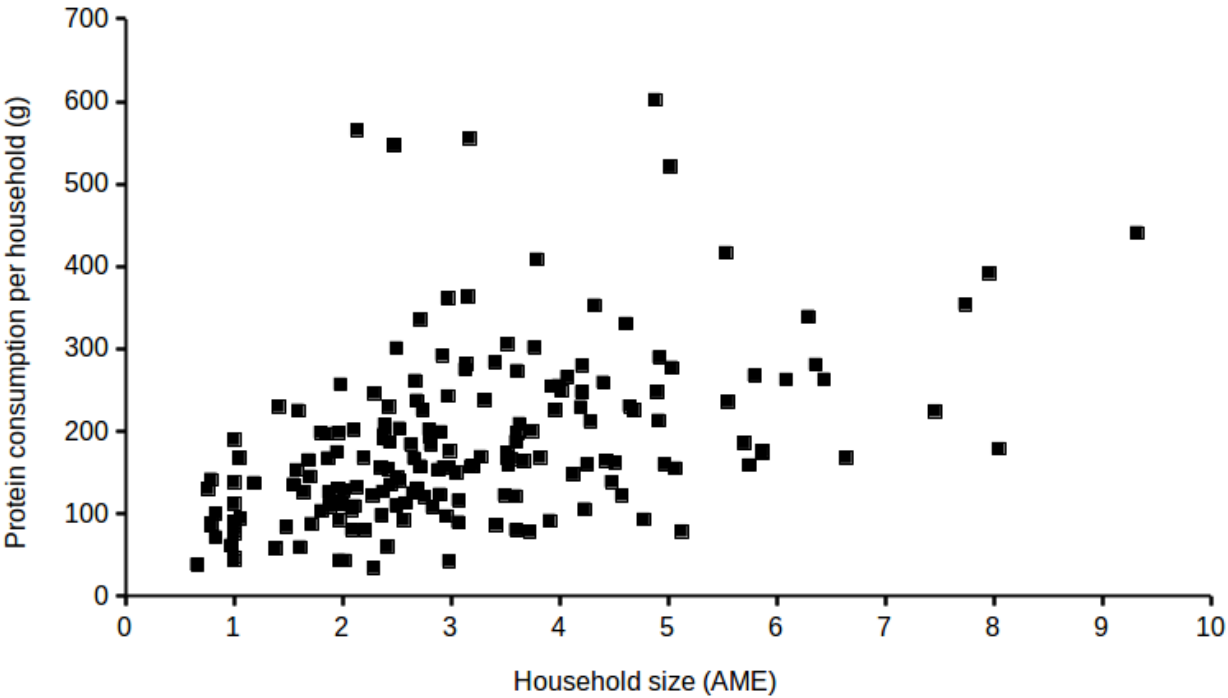

Supplement: S1 Fig — (PDF) [file pone.0188109.s007.pdf]
