# Supplementary material for: Rural protein insufficiency in a wildlife-depleted West African farm-forest landscape
Source: PLoS One. 2017 Dec 13;12(12):e0188109. doi: 10.1371/journal.pone.0188109 (PMC5728563; doi:10.1371/journal.pone.0188109)

S2 Fig. Effect of households wealth rank on household size (GLMM results in Table S3). Standard errors are shown.

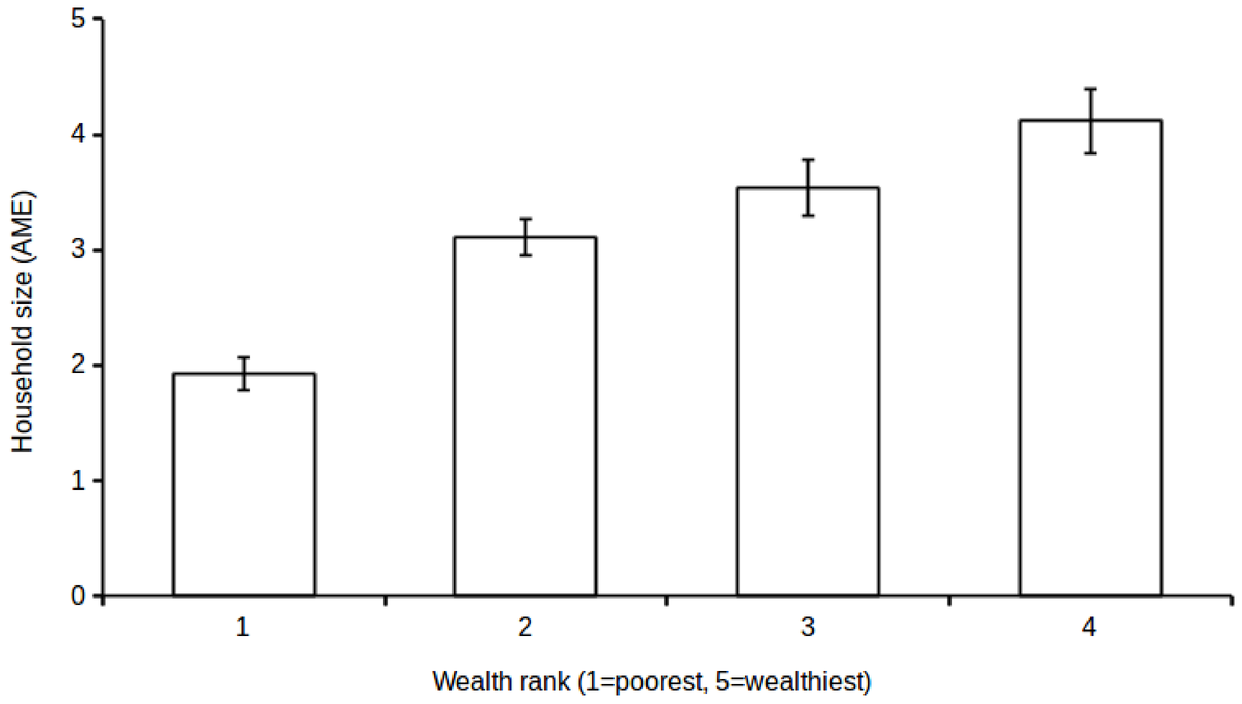

Supplement: S2 Fig — Standard errors are shown. (PDF) [file pone.0188109.s008.pdf]
