# Supplementary material for: Rural protein insufficiency in a wildlife-depleted West African farm-forest landscape
Source: PLoS One. 2017 Dec 13;12(12):e0188109. doi: 10.1371/journal.pone.0188109 (PMC5728563; doi:10.1371/journal.pone.0188109)
Supplement: S1 Questionnaire — (PDF) [file pone.0188109.s009.pdf]

HH SURVEY	Date:	Time start:	Time stop:	Original / Copy	HhID:	SheetID: Hh0	Problem:
Interviewee name/PersID:		Observer:	Sheet checked by:		Inputted by:	Input checked by:	

T1	PRODUCTION<br>Product name	# unit	UnitID	#/unit	Size	Tot. KG	Total WSP price	Collector PersID	Carrier PersID	From where		Purpose %					FarmID
										Location	LandTyp	Cons	Sale	Proc	Gift	Plant	
1																	
2																	
3																	
4																	
5																	
6																	
7																	
8																	
9																	
10																	
11																	

T2	EXPENDITURE<br>Expenditure type	Food	Meal	Item	Work	Service	Planted	24hr	Origin P/G/B	# units	Unit name	Tot. Kg	Total value	Credit	Lender PersID	Who paid PersID	Recipient PersID	Location	Purpose %				
																			Cons	Sale	Proc	Gift	Plant
1																							
2																							
3																							
4																							
5																							
6																							
7																							
8																							
9																							
10																							
11																							

T3	INCOME (Labour)<br>Labour details	Worker PersID	Employer PersID	Employer Work Rel.	Recipient PersID	Hrs worked	Salary/chop (old cedi)	Crop salary value	Work location	Food provided	Provider PersID	Value of food
1												

T4	**INCOME (RawProd)** Sold product type	# units	unit name	Size	Tot. Kg	Total Sales price	Origin P/G/B	24hr	Collector PersID	Collected where: Location	Collected where: LandType	SoldBy PersID	SoldTo PersID	Location sale	Transp. cost	Market cost
1																
2																
3																

T5	**GIFTS** Gift details	Income	Expend	Food	Meal	Item	Work	Money	Plant	From PersID	Recipient PersID	# units	unit name	Weight (kg)	Total Amount / WSP price	24hr	Origin P/G/B	Purpose %: Cons	Sale	Proc	Gift	Plant
1																						
2																						
3																						
4																						
5																						
6																						
7																						
9																						

T6	**MEAT CONSUMP**	# meat meals			Eaten at Hh or bought & eaten			Origin this %		
	Type of meat	D	B	L	# HhMem	# NonHhM	cedis this / other Hh	P	G	B
1										
2										
3										
4										

T7		# meat meals			Meat eaten outside	
	Type of meat	D	B	L	# HhMem	cedis this / other Hh
1						
2						
3						
4						

LAST NIGHT'S DINNER SURVEY – Hh & Non-Hh members consuming dinner at this house

T8	Eater Name	PersID	HhMem
1			
2			
3			
4			

	Eater Name	PersID	HhMem
5			
6			
7			
8			

	Eater Name	PersID	HhMem
9			
10			
11			
12			

	Eater Name	PersID	HhMem
13			
14			
15			
16			

Food consumed outside this house by Hh members

T9	Eater Name	PersID	HhID
1			
2			
3			

	Eater Name	PersID	HhID
5			
6			
7			

	Eater Name	PersID	HhID
8			
9			
10			

	Eater Name	PersID	HhID
11			
12			
13			

T 10	**HUNT/TRAP/FISH** Animal name	24hr	2Wk	Hunter PersID	# Animals	Size	Method G/T/D/H	Location	Land Type	Edge	Time M/A/E/N	Total WSP price	Take gun	Check traps	Costs (bullet/wire)	Other activity	% Purpose Cons	Sale	Proc	Gift
1																				
2																				
3																				
4																				
5																				
6																				

T 11	**INCOME (Non-Raw)** Product name	End	Input	# units	Unit ID	Total price	Origin P/G/B	SoldBy PersID	SoldTo PersID	Location	Transport cost	Market cost
1												
2												
3												
4												
5												
6												

T 12	**EXPENDITURE/GIFT** **> 50,000 last 2 weeks**	Buy	Gift	Lend	Food	Meal	Item	Work	Money	Service	Credit	Lender PersID	# units	unit name	Total Value	Value per term	Who paid PersID	Recipient PersID	Location
1																			
2																			
3																			
4																			
5																			
6																			
7																			
8																			

T 13	**INCOME/GIFT** **> 50,000 last 2 weeks**	Income	Gift	Borrow	Food	Meal	Item	Work	Money	Service	Credit	Lender PersID	# units	unit name	Total Value	Who paid PersID	Recipient PersID	Location
1																		
2																		
3																		
4																		
5																		

Expenditures at market within last week that are not covered by 24hr recall

T	**Markets not last 24hr**	Income	Expend	Market ID			Food	Meal	Service	Item	Car	Market	#	unit name	Total	Who paid	Who sold	% Purpose			
14	Product			DW	NS	Other							units		Value	PersID	PersID	Cons	Sale	Proc	Gift
1																					
2																					
3																					
4																					
5																					
6																					
7																					
8																					
9																					
10																					
11																					
12																					
13																					
14																					

Comments:
